# Exopolysaccharides from *Bifidobacterium animalis* Ameliorate *Escherichia coli*-Induced IPEC-J2 Cell Damage via Inhibiting Apoptosis and Restoring Autophagy

**DOI:** 10.3390/microorganisms9112363

**Published:** 2021-11-16

**Authors:** Lanxin Yuan, Bingxin Chu, Shiyan Chen, Yanan Li, Ning Liu, Yaohong Zhu, Dong Zhou

**Affiliations:** 1 College of Veterinary Medicine, China Agricultural University, Beijing 100193, China; yuanlanxin914@163.com (L.Y.); barrylao@163.com (B.C.); chen_shiyan1993@163.com (S.C.); 13796685756@163.com (Y.L.); nliu2224@163.com (N.L.); 2 College of Veterinary Medicine, Northwest A&F University, Xianyang 712100, China

**Keywords:** *Bifidobacterium animalis* subsp. *lactis*, exopolysaccharide, IPEC-J2, autophagy

## Abstract

Enteropathogenic *Escherichia coli* (EPEC) is a common zoonotic pathogen that causes acute infectious diarrhea. Probiotics like *Bifidobacterium* are known to help prevent pathogen infections. The protective effects of *Bifidobacterium* are closely associated with its secretory products exopolysaccharides (EPS). We explored the effects of the EPS from *Bifidobacterium animalis* subsp. *lactis* (*B. lactis*) on ameliorating the damage of an intestinal porcine epithelial cell line (IPEC-J2) during EPEC infection. Pretreatment with EPS alleviated EPEC-induced apoptosis through the restoration of cell morphology and the downregulation of protein expressions of cleaved-caspase 8, cleaved-caspase 3, and cleaved-PARP. EPS-mediated remission of apoptosis significantly improved cell viability during EPEC infection. EPEC infection also resulted in impaired autophagy, as demonstrated by decreased expressions of autophagy-related proteins Beclin 1, ATG5, and microtubule-binding protein light chain-3B (LC3B) and the increased expression of p62 through western blot analysis. However, EPS reversed these effects which indicated that EPS promoted autophagosome formation. Furthermore, EPS prevented the lysosome damage induced by EPEC as it enhanced lysosomal acidification and raised lysosome-associated protein levels, thus promoted autophagosome degradation. Our findings suggest that the amelioration of EPEC-induced cell damages by EPS is associated with the limitation of detrimental apoptosis and the promotion of autophagy flux.

## 1. Introduction

Enteropathogenic *Escherichia coli* (EPEC) is a Gram-negative bacterium that spreads via oral-fecal contact with contaminated hands or foods [1]. It adheres to the mucosa of the intestines, resulting in acute and persistent watery diarrhea in humans and animals worldwide [2]. EPEC is a major *E. coli* pathotype that is estimated to be responsible for piglet postweaning diarrhea in China, and a total of 94.15% of 171 *E. coli* isolates from 56 swine farms were multidrug-resistant [3,4]. A new strategy is necessary for the prevention of pathogen infections since antimicrobial resistance becomes general [5]. 

*Bifidobacterium* has been demonstrated to help prevent gastrointestinal infections [6,7]. Treatment with *Bifidobacterium longum* subsp. *Infantis* and *Bifidobacterium animalis* subsp. *lactis* (*B. lactis*) reduce the pathogen load in the colon and feces and ameliorate ileal damage caused by *Salmonella* Typhimurium [8]. *B. lactis* also reduce the adherent-invasive *Escherichia coli* adhesion and alleviate inflammation within intestinal epithelial cells [9].

The beneficial functions may be related to secretory products especially exopolysaccharides (EPS) [10]. Bacterial EPS are partly secreted into the environment as secondary metabolic products and partly covered the bacterial surface [11] and can be classified into homopolysaccharides and heteropolysaccharides according to the monomer composition and mechanisms of biosynthesis [12]. The reported EPS from *Bifidobacterium* are all heteropolysaccharides composed of galactose, glucose, and rhamnose, among others [13]. EPS is one of the potential substitutes for antibiotics. It is hypothesized that the protective mechanism of EPS may be the formation of a “biofilm-like” layer on the intestinal epithelium that prevents the adhesion of pathogens or their toxins [14]. EPS from *Bifidobacterium bifidum* WBIN03 exhibit antibacterial activities against *E. coli* [15]. Nevertheless, few studies have been conducted to deeply explore the protective mechanism of EPS. 

Intestinal epithelial cells represent the first line of interaction between host and pathogens in the gut mucosa [9]. After entering the gastrointestinal tract, EPEC adheres to the intestinal epithelial cells and disturbs intracellular signaling pathways by injecting virulence factors [16]. EPEC effector protein causes mitochondrial dysfunction leading to host cell apoptosis which potentially aggravates intestinal infection [2]. In the process of apoptosis, caspase-8 is an apical caspase that can trigger the activation of downstream effector caspases such as caspase-3 after cleavage, then proteolytic activation of caspase-3 and PARP eventually stimulating cell death [17,18]. Caspase-8, caspase-3, and PARP are activated in EPEC-infected human intestinal epithelial cells [19]. EPS from *Bifidobacterium breve* reduce cleaved caspase-3 mediated epithelial cell shedding induced by lipopolysaccharide from *E. coli* in the ileum [20]. *Bifidobacterium infantis* conditioned medium attenuates apoptosis of epithelium cells in *Cronobacter sakazakii*-infected ileum [21]. However, the role of EPS in apoptosis induced by *E. coli* is unclear.

Autophagy serves as a major protective process that maintains intestinal homeostasis to adapt to various stresses such as microbial infection [22]. Autophagy is characterized by the formation of a double-membrane autophagosome that involves Beclin 1, several ATG proteins, and LC3B which is a commonly used autophagosome marker. The autophagosomes fuse with lysosomes, resulting in the degradation of engulfed components [23]. *E. coli* suppresses autophagy to evade host defense and facilitate adhesion on intestinal epithelial cell surfaces [24]. Supernatants collected from four types of Bifidobacteria trigger autophagy response in intestinal epithelial cells [25]. *Bifidobacterium longum* subsp. *Infantis* promoted autophagy in *Salmonella*-infected Caco-2 cells, thereby leading to the clearance of pathogens [26]. Whether administration of EPS from *B. lactis* contributes to EPEC clearance via autophagy remains to be determined.

Hereby, this research was designed to assess whether the administration of EPS from *B. lactis* had a protective effect against EPEC-induced intestinal damage *in vitro*. We established an EPEC infection model using IPEC-J2 cells to assess apoptosis-associated cell damage and autophagy markers, thereby testing the hypothesis that EPS from *B. lactis* attenuates EPEC-induced cell damage by inhibiting apoptosis and restoring autophagy.

## 2. Materials and Methods

### 2.1. Cell Culture and Treatment

IPEC-J2 cells were grown in Dulbecco’s Modified Eagle Medium/Ham’s F-12 medium (1:1) (Cytiva, Marlborough, MA, USA) supplemented with 10% fetal bovine serum (ThermoFish Scientific, Waltham, MA, USA) at 37 °C with 5% CO_2_. An antibiotic mixture (100 U/mL penicillin and 100 μg/mL streptomycin, Invitrogen, Carlsbad, CA, USA) was added to the medium for bacteria-free assays [27]. For bacterial infections, cells were seeded at a density of 10^6^ cells per well in 6-well culture plates then treated with medium, EPEC (5 × 10^7^ CFU, multiplicity of infection, MOI = 50), EPS (5 μg) respectively, or preincubated with EPS (5 μg) for 2 h before EPEC infection (EPEC + EPS). Cells were washed three times by PBS after the preincubation with EPS then infected with EPEC (5 × 10^7^ CFU) for 8 h. An autophagy inhibitor Chloroquine (CQ, C6628, Sigma-Aldrich, St. Louis, MO, USA) was applied to the experiment. In detail, 50 μM CQ was added into the medium 1 h before co-incubated with EPS for 2 h, then cells were harvested.

### 2.2. Bacterial Strains and Preparation of Conditioned Media

*Bifidobacterium animalis* subsp. *lactis* was isolated from feces of healthy pigs and it was grown in De Man, Rogosa, and Sharpe (MRS, Oxoid, Hampshire, UK) broth supplemented with 0.2% L-cysteine and 5% equine serum for 36 h at 37 °C under the anaerobic condition. *Escherichia coli* strain O111:K58 (CVCC1450; China Institute of Veterinary Drug Center, Beijing, China) was grown in Luria-Bertani (LB, Oxoid, Basingstoke, England) broth at 37 °C with constant shaking, until reaching the mid-log phase [28].

### 2.3. Extraction, Purification, and Detection of EPS

*Bifidobacterium animalis* subsp. *lactis* was inoculated (5%) in 500 mL MRS described above for 36 h at 37 °C under the anaerobic condition. The supernatant was collected after centrifugation (4000× *g*, 10 min), then the pH of the supernatant was adjusted to 7.5 and added 0.25% trypsin (supernatant/trypsin 4:1) for 1.5 h at 45 °C to remove proteins. After that, trypsin was inactivated by heat. For extraction, the supernatant was precipitated with a triploid volume of ethanol overnight at 4 °C followed by centrifugation (8000× *g*, 10 min). The precipitates were collected and dissolved in distilled water. Afterward, a double volume of Sevage solution (chloroform/n-butanol 4:1) was used for deproteinization. In the end, the collected EPS was dialyzed against distilled water for 2 days at 4 °C and can be applied to experiment after vacuum freeze-drying. The concentration of EPS was measured by using the phenol-sulfuric acid assay method [15]. 

### 2.4. Cell Viability Assay

Cell counting kit-8 (Solarbio, Beijing, China) was used to determine cell viability following instructions. Cells were plated in a 96-well plate at a density of 10^4^ cells per well then treated the same way as described before. CCK-8 solution was added to the culture medium at the end of the treatment and incubated for 1 h at 37 °C. Microplate Reader (Bio-Rad, Hercules, CA, USA) was used to detect the optical density at 450 nm [29]. 

### 2.5. Calcein-AM/PI Staining

Cells were plated in a 24-well plate at a density of 2.5 × 10^5^ cells per well then treated the same way as described above. Calcein-AM/PI assay buffer was prepared and added to the culture medium then incubated for 30 min at 37 °C following the instruction. Slides were placed and observed under a laser scanning confocal microscope with excitation set at 488 nm to view both living cells (green fluorescence) and dead cells (red fluorescence) while at 545 nm could only view dead cells. 

### 2.6. MDC Staining

Dansylcadaverine (MDC) is a fluorescent stain, which is usually used to detect autophagosome formation. Cells were plated on sterile coverslips placed in 24-well plates at a density of 2.5 × 10^5^ cells per well then MDC staining solution was added and incubated for 30 min under normal growth conditions to label the autophagosome. Slides were washed with wash buffer which is equipped in the kit three times and fixed with 4% paraformaldehyde for 10 min, edges were sealed then specimens were examined with laser scanning confocal microscope afterward.

### 2.7. Lyso-Tracker Red Staining

Cells were plated on sterile coverslips placed in 24-well plates at a density of 2.5 × 10^5^ cells per well then lyso-tracker red staining solution was added and incubated for 2 h under normal growth conditions to label lysosomes. Slides were washed with PBS twice and fixed with 4% paraformaldehyde for 10 min, edges were sealed then specimens were examined with laser scanning confocal microscope afterward.

### 2.8. Acridine Orange Staining

Cells were grown in 24-well plates at a density of 2.5 × 10^5^ cells per well then 13 μg/mL acridine orange staining solution was added and incubated for 20 min under normal growth conditions. Cells were washed with warm PBS twice and examined with laser scanning confocal microscope afterward with excitation at 488 nm. Green fluorescence (emission peak between 530 and 550 nm) and red fluorescence (emission peak at about 650 nm) were simultaneously collected by two separate windows.

### 2.9. Western Blotting

Proteins were analyzed by western blotting. The following primary antibodies were purchased from ProteinTech Group (Rosemont, IL, USA): anti-caspase-8 (1:750, 13423-1-AP), anti-PARP (1:500, 13371-1-AP), anti-p62 (1:1500, 18420-1-AP), anti-ATG 5 (1:750, 10181-2-AP), anti-Beclin 1 (1:1000, 11306-1-AP), anti-ATP6V1A (1:1000, 17115-1-AP), anti-ATP6V1B2 (1:2000, 15097-1-AP), anti-ATP6V1E1 (1:3000, 15280-1-AP), anti-Cathepsin B (1:500, 12216-1-AP), anti-Cathepsin D (1:2000, 21327-1-AP). Anti-β-actin antibody (1:5000, 66009-1-Ig) was used to confirm the consistency of sample loading. Anti-cleaved caspase-3 (1:1000, 9664) and anti-LC3A/B (1:1000, 12741) were purchased from Cell Signaling Technology (Danvers, MA, USA). Anti-LAMP1 (1:500, WL02419) was purchased from Wanleibio (Shenyang, China). Besides, Horseradish peroxidase-conjugated AffiniPure goat anti-mouse IgG (1:5000, SA00001-1) or goat anti-rabbit IgG (1:5000, SA00001-2) from ProteinTech Group (Rosemont, IL, USA) were used as secondary antibodies [27].

### 2.10. Statistical Analysis

All experiments were repeated three times. Statistical analysis (ANOVA) was performed and visualized using GraphPad Prism 6.0 software (Graphpad Software Inc., San Diego, CA, USA) while *p* < 0.05 was considered statistically significant. All results were expressed as means ± SD deviation.

## 3. Results

### 3.1. Pretreatment with EPS Reduced the Cell Death Induced by EPEC

**Figure 1:**
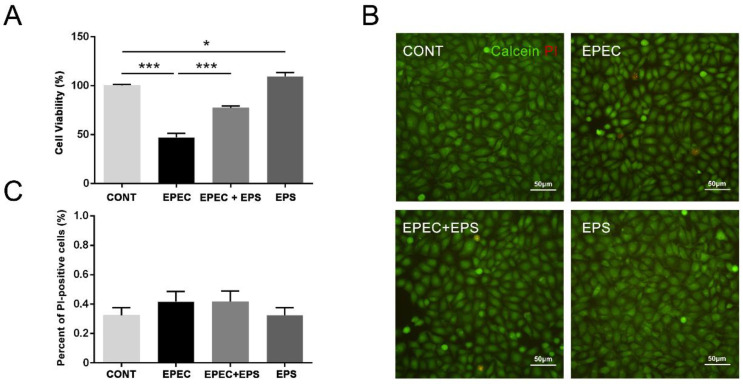
Pretreatment with EPS reduced the cell death induced by EPEC. (**A**) Cell viability was detected through the CCK-8 assay. CCK-8, Cell Counting Kit-8; The data was presented as mean ± SEM (*n* = 3), * *p* < 0.05, *** *p* < 0.001. (**B**) Representative image of IPEC-J2 cells stained by Calcein-AM/PI was showed. Scale bar, 50 µm. (**C**) The percentage of PI-positive cells was presented as mean ± SEM (*n* = 3), there was no difference among four groups.

Compared with the control group, cell viability was significantly decreased after EPEC infection, while pre-incubation of EPS could effectively reduce the cell mortality (Figure 1A). Besides, EPS treatment significantly increased cell viability, suggesting that EPS can promote cell growth to some extent. Thus, these data demonstrated that EPS alleviates EPEC-induced cell viability inhibition in IPEC-J2 cells. We then used calcein-AM/PI staining to double fluorescent labeling the living cells and dead cells. Calcein-AM can label living cells (green fluorescence), while PI can only label dead cells (red fluorescence) through the damaged cell membrane. As shown in Figure 1B,C, although EPEC reduced the viability of IPEC-J2 cells by 50% (Figure 1A), it did not change the permeability of the cell membrane.

### 3.2. Pretreatment with EPS Alleviated EPEC-Induced Cell Apoptosis in IPEC-J2 cells

**Figure 2:**
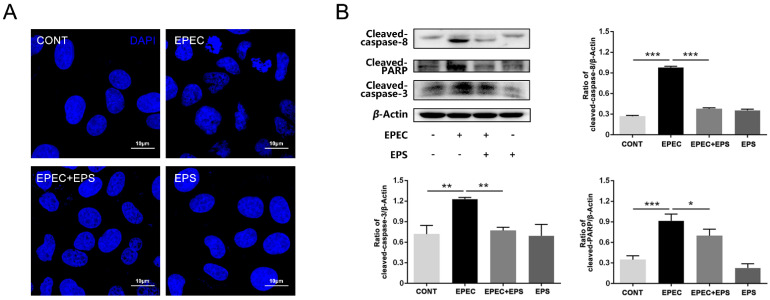
Pretreatment with EPS alleviated EPEC-induced cell apoptosis in IPEC-J2 cells. (**A**) Detection of the apoptotic morphological changes in IPEC-J2 cells by DAPI staining. Scale bars, 10 µm. (**B**) Western blot analysis for the protein levels of cleaved-caspase-8, cleaved-PARP, and cleaved-caspase-3 in IPEC-J2 cells and surrounding panel was the quantitation analysis by Image J software. The data was presented as mean ± SEM (*n* = 3), * *p* < 0.05, ** *p* < 0.01, *** *p* < 0.001.

Apoptosis contributed to EPEC-induced cell death since morphological analysis showed obviously chromatin condensation, nuclear shrinkage, and fragmentation in EPEC-infected cells (Figure 2A). Further, the western blot analysis revealed that the protein levels of cleaved-caspase 8, cleaved-caspase 3, and cleaved-PARP significantly increased after EPEC treatment (*p* < 0.01) (Figure 2B). These results confirmed that EPEC infection induced apoptosis in IPEC-J2 cells. But pretreatment with EPS alleviated the cell damage (Figure 2A). Western blot analysis revealed that there was a significant decrease in cleaved-caspase 8, cleaved-caspase 3, and cleaved-PARP protein expressions in the EPEC + EPS group compared with the EPEC group (*p* < 0.05) (Figure 2B). Results of apoptosis morphology and western blot showed that EPS could reduce apoptosis induced by EPEC.

### 3.3. EPS restored the Autophagy Flux Inhibited by EPEC in IPEC-J2 Cells

**Figure 3:**
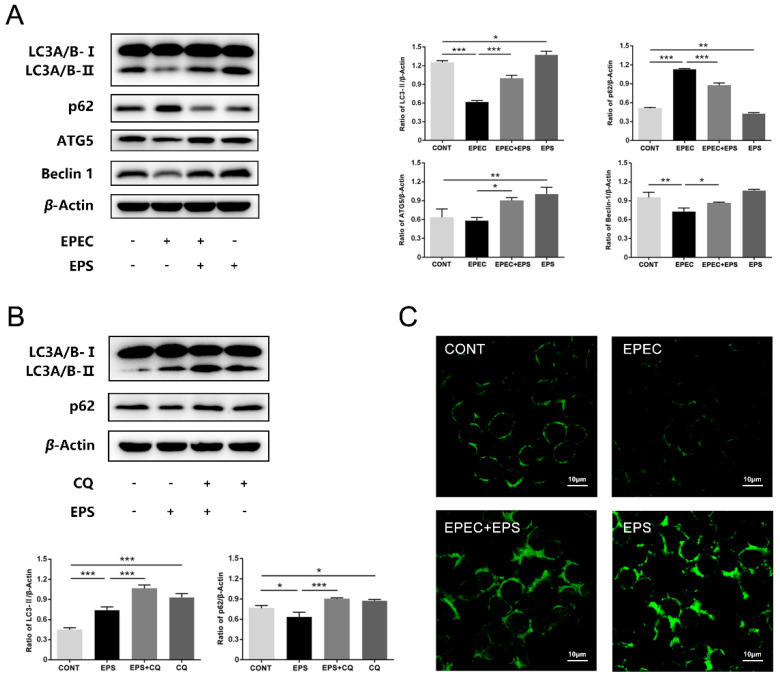
EPS restored the autophagy flux inhibited by EPEC in IPEC-J2 cells. (**A**) The protein expressions of LC3, p62, ATG5, and Beclin 1 in IPEC-J2 cells and right panels were the quantitation analysis by Image J software. (**B**) CQ was added 1 h before co-incubated with EPS for 2 h to inhibit autophagic degradation, western blot analysis for the protein expressions of LC3 and p62 in IPEC-J2 cells was showed, and the panel below was the quantitation analysis by Image J software. (**C**) Autophagosome formation was assessed by MDC staining. Representative autophagosome formation of autophagy was presented. Scale bars, 10 µm. The data was presented as mean ± SEM (*n* = 3), * *p* < 0.05, ** *p* < 0.01, *** *p* < 0.001.

During the process of autophagy, LC3B-I is converted to LC3B-II by phosphatidylethanolamine conjugation. Thus, tracking the change of LC3B-II is indicative of autophagic activity [23]. Western blot analysis showed that there was a significant decrease in protein levels of LC3B-II and Beclin 1 and a significant increase in p62 protein expression after EPEC challenged compared with the control group (*p* < 0.01) which indicated that autophagic flux was impaired [30] in EPEC-infected IPEC-J2 cells (Figure 3A). Consistent with this finding, MDC staining showed that green fluorescence in the EPEC group was less than that in the control group (Figure 3C). However, the protein expressions of LC3B-II, ATG5, and Beclin 1 were increased and that of p62 was decreased in the EPEC + EPS group compared with the EPEC group (*p* < 0.05) which indicated that EPS can alleviate EPEC-induced cell autophagy inhibition via enhancement of autophagy (Figure 3A). In order to further confirm that EPS can enhance autophagy, CQ was applied to block the downstream steps of autophagy. The western blot analysis showed that LC3B-II and p62 expression in the EPS + CQ group significantly increased compared with the EPS group (*p* < 0.001) (Figure 3B). MDC staining showed that compared to the EPEC group, the EPEC + EPS group showed more green fluorescence (Figure 3C). Therefore, we concluded that EPS could stimulate autophagy flux inhibited by EPEC through increasing autophagosomes formation and accelerating autophagosome clearance.

### 3.4. EPS Resisted Lysosomal Alkalization Caused by EPEC

**Figure 4:**
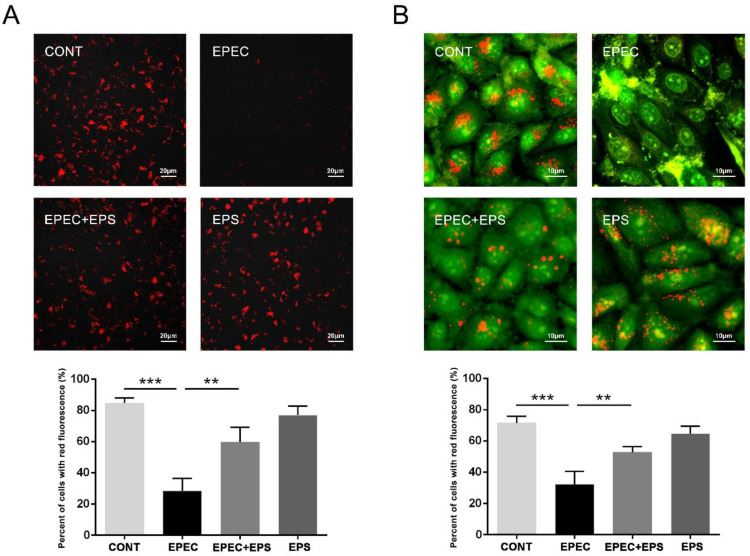
EPS resisted lysosomal alkalization caused by EPEC. (**A**) Lyso-Tracker Red staining was used to assess the lysosome acidity in IPEC-J2 cells. Scale bars, 20 µm. The panel below was the percentage of cells with red fluorescence by Image J software. (**B**) Acridine orange staining was also used to assess the lysosome acidity in IPEC-J2 cells. Scale bars, 10 µm. The panel below was the percentage of cells with red fluorescence by Image J software. The data was presented as mean ± SEM (*n* = 3), ** *p* < 0.01, *** *p* < 0.001.

Of note, autophagosomes need to combine with lysosomes to degrade and recycle substrates. EPEC inhibited the degradation of autophagosomes, so we speculated that EPEC might affect the normal function of lysosomes. We then evaluated whether the effect of EPS to stimulate autophagic flux was related to the restoration of lysosomal function. Two sensitive lysosomotropic pH probes were applied to evaluate the effect of EPEC on lysosomal pH in IPEC-J2 cells. Lyso-Tracker Red (LTR) manifests red fluorescence in a pH-dependent manner in the lysosome and the increased staining indicates the reduced lysosomal pH. EPEC significantly abolished the LTR staining due to lysosomal alkalinization in IPEC-J2 cells compared with the control group while the EPEC + EPS group increased LTR staining (enhanced lysosomal acidification) compared with the EPEC group (Figure 4A). AO staining presents green fluorescence in the cytosol but red fluorescence in the acidic compartments. Thus, a decrease in granular red fluorescence with an increase in diffuse green fluorescence implies an elevated lysosomal pH. The data further verified that EPEC caused lysosomal alkalinization in IPEC-J2 cells and EPS can alleviate this alkalinization (Figure 4B).

### 3.5. EPS Alleviated Lysosomal Damage Caused by EPEC in IPEC-J2 Cells

**Figure 5:**
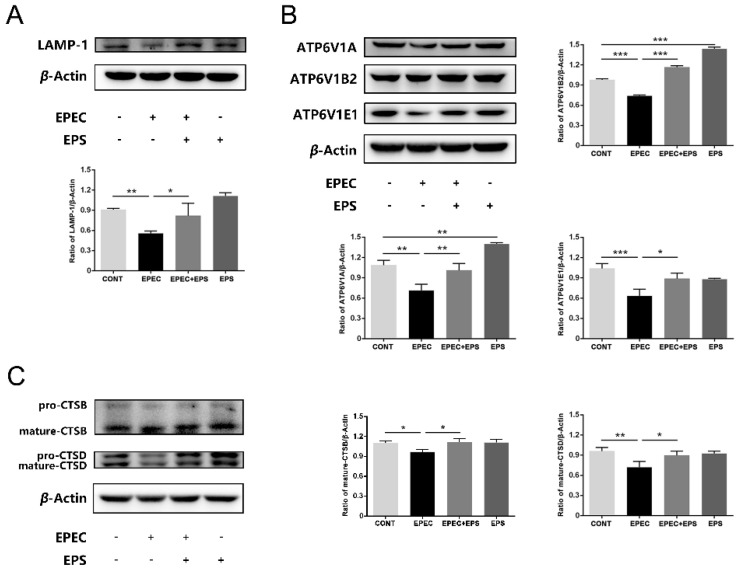
EPS alleviated lysosomal damage caused by EPEC in IPEC-J2 cells. (**A**) Western blot analysis for the protein expression levels of LAMP-1. (**B**) Western blot analysis for the protein expression levels of ATP6V1A, ATP6V1B2, and ATP6V1E1. The panel below showed the quantitation analysis by Image J software. (**C**) Western blot analysis for the protein expression levels of CTSB and CTSD in IPEC-J2 cells and the right panel was the quantitation analysis by Image J software. The data was presented as mean ± SEM (*n* = 3), * *p* < 0.05, ** *p* < 0.01, *** *p* < 0.001.

**Figure 6:**
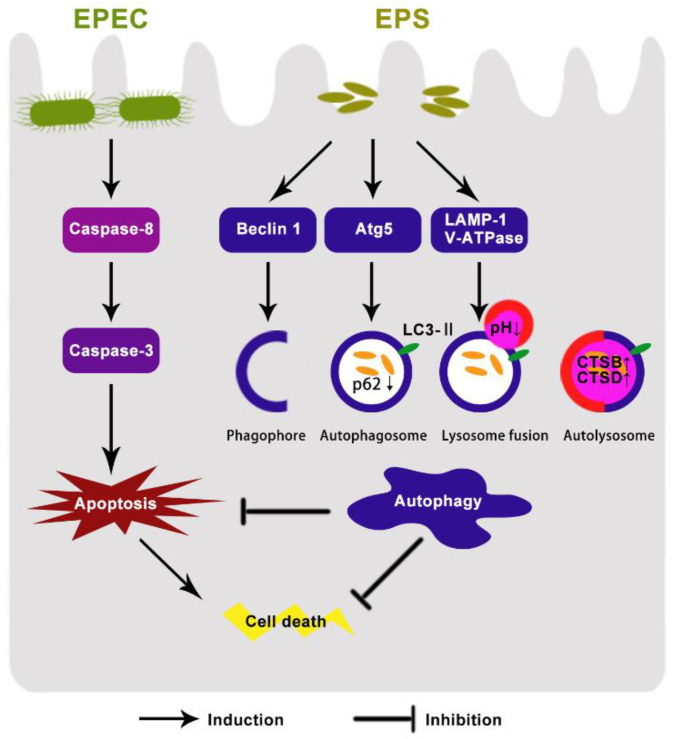
Proposed model of the protective effect of *B. lactis* EPS in EPEC-induced cell damage. EPEC results in impaired autophagy and apoptosis. *B. lactis* EPS promotes autophagosome formation by up-regulating the expressions of proteins Beclin 1, ATG5 and LC3-II and enhances lysosomal function to accelerate autophagosome degradation. *B. lactis* EPS inhibits apoptosis via decreasing the expressions of the apoptotic proteins caspase-8 and caspase-3. Through this mechanism, *B. lactis* EPS protects against EPEC-induced apoptotic cell death.

We assumed that EPEC might disturb the function of lysosomes. Western blotting assay results showed that compare with the control group, EPEC downregulated the expression of glycosylated membrane structural proteins LAMP1, but this decrease was alleviated by EPS (*p* < 0.05) (Figure 5A). Since lysosome acidification is partially correlated with the activity of vacuolar ATPase (V-ATPase), changes in protein levels of three V-ATPase subunits located in lysosomes of IPEC-J2 cells were assessed in this study. Compared with the control group, there was a decrease in ATP6V1A, ATP6V1B2, and ATPV1E1 protein levels in EPEC infected cells while the EPEC + EPS group increased significantly in all V-ATPase subunits compared with the EPEC group (Figure 5B) (*p* < 0.05). Cysteine protease cathepsin B (CTSB) and aspartic protease cathepsin D (CTSD) are the most abundant lysosomal proteases. To examine how EPEC might affect the lysosomal function, intracellular protein levels of CTSB and CTSD in IPEC-J2 cells were assessed by western blotting analysis. Consistently, EPEC significantly impaired the maturation of CTSB and CTSD (Figure 5C) (*p* < 0.05) but EPS can significantly reverse this impairment (*p* < 0.05). These data indicated that EPEC impaired lysosomal function and EPS can alleviate the impairment by promoting V-ATPase subunits and cathepsin expression level. The proposed model of the protective effect of *B. lactis* EPS in EPEC-induced cell damage was shown in Figure 6.

## 4. Discussion

*Bifidobacterium* is a key player in intestinal microbiology and gut immunology. The health-promoting effects of *Bifidobacterium* are suggested to be related to the biological activities of EPS [13]. EPS is thought to adhere to the cell surface via its glycosylated and interact with proteins or peptides, and the conjugation may be recognized by transmembrane proteins and further elicit specific cell signaling [11].In this study, we reported that EPEC induced apoptosis and inhibited autophagy in IPEC-J2 cells. Pretreatment with EPS from *B. lactis* significantly reduced cell death caused by EPEC via alleviating apoptosis and restoring autophagy flux, which provides valuable insights into the mechanisms of *B. lactis* in maintaining gut health.

Apoptosis plays a central role in host-pathogen interactions. During infection, *E. coli* induces upregulation of apoptosis and leads to intestinal barrier dysfunction, thereby triggering diarrhea in piglets [31]. We found that EPEC infection activated apoptosis and decreased the viability of IPEC-J2 cells. The treatment with EPS prevented excessive cell death, restored the destruction of cell morphology, and significantly suppressed apoptosis indicated by inhibiting protein expressions of cleaved caspase-8, cleaved caspase-3, and cleaved PARP. These data strongly suggested that EPS may block apoptosis signaling to protect epithelial cells during infection. EPS from *Bifidobacterium breve* attenuated apoptosis induced by LPS from *E. coli* in ileum indicated by reducing the level of cleaved caspase 3-positive shedding cells remarkably [20]. In the necrotizing enterocolitis model, *Bifidobacterium infantis* and *Bifidobacterium bifidum* were shown to alleviate apoptosis, thus reduced mucosal injury and preserved intestinal integrity in the ileum [21,32]. However, there is a potential risk that high concentration and long processing time of EPS from lactic acid bacteria may induce cell apoptosis instead [33].

When cells are exposed to stress signals like infections, the autophagy that arises from the inhibition of apoptosis actually protects cells from death. Autophagy inhibits apoptosis by selectively removing apoptosis-stimulating factors and maintains homeostasis [34]. *Bacillus amyloliquefaciens* SC06-induced autophagy contributes to the suppression of apoptosis thus protected macrophages against *E. coli* infection [35]. Since EPS alleviated apoptosis caused by EPEC, we then explored the effect of EPS on induction of autophagy. 

Autophagy acts as a microbial surveillance and clearance system in the epithelium [36]. During the initiation of autophagy, Beclin 1 is part of the complex that plays a key role in phagophore nucleation [37]. Then, autophagosome formation is mediated by the ATG5-ATG12 conjugation system and the LC3 processing system [23]. *E. coli* facilitates pathogen colonization and growth by subverting autophagy in human colonic epithelial cells via downregulating ATG5 and LC3B-II protein levels [24]. Consistent with our study, the protein levels of Beclin 1, ATG5, and LC3B-II were decreased during EPEC infection, whereas EPS upregulated these protein expressions, suggesting that EPS promoted autophagosome synthesis. Notably, the EPS-producing strain *Streptococcus thermophilus* BGKMJ1-36 was proved that it can trigger autophagy indicated as significantly upregulating the expressions of autophagy-related genes including Beclin 1, ATG5, and LC3B-II [38]. A previous study showed that *Lactobacillus johnsonii* L531 increased ATG5 protein expression for maintaining the autophagy process to accelerate *Salmonella* infantis clearance in IPEC-J2 cells [27].

Moreover, EPEC infection upregulated the expression level of p62 and this increase has been considered as an indicator of blocked autophagy flux, especially at the stage of lysosome-mediated autophagic degradation [30]. Preincubation with EPS downregulated p62 protein expression which indicated that autophagy flux was promoted and the elevated LC3B-II protein expression level was not due to the inhibition of autophagy degradation. Furthermore, cells pretreated with CQ, an inhibitor that impairs autophagic degradation, facilitated the accumulation of LC3B-II and p62 during EPS treatment. Therefore, we concluded that EPS could not only promote autophagosome synthesis but also accelerate autophagosome degradation to help maintain homeostasis in IPEC-J2 cells during EPEC infection.

We then paid attention to the degradation process of autophagy, especially the function of the lysosome. The lysosome is crucial to the degradation process of autophagy as it provides the hydrolases and the acidic pH necessary to degrade the autophagic substrates [39]. We used two sensitive lysosomotropic pH probes to detect lysosomal pH in IPEC-J2 cells. Results showed that EPEC caused lysosomal alkalinization, while EPS alleviated alkalinization by reducing lysosomal pH. The acidic pH of the lysosomal lumen is maintained by the V-ATPase embedded in the limiting membrane [40]. We found that EPEC perturbed lysosomal pH by inhibiting V-ATPases (ATPV1A, ATPV1B2, and ATPV1E1) activity. EPS enhanced the activity of V-ATPases, thereby increased lysosomal acidification. Our results also showed that preincubation with EPS improved the expression level of LAMP-1, a crucial protein associates with the lysosomal membrane to help establish or maintain its physical integrity [41], and to influence autolysosome maturation. Within the lysosome, CTSB and CTSD are necessary for the maturation of the autolysosome [40]. EPS promoted the maturation of CTSB and CTSD thereby strengthened the degradation ability of lysosomes. Thus, we concluded that EPS could strengthen the degradation ability of lysosomes and then promote the degradation process of autophagy. *Lactobacillus paracasei* supernatant significantly advanced lysosome fusion to reduce the viability of celiac disease-associated *Neisseria flavescens* in Caco-2 epithelial cells [42].

This study provided a new theoretical basis for the application of EPS in the prevention of bacterial diarrhea. The IPEC-J2 model provided an in vitro framework for evaluating EPS intervention in diarrhea induced by EPEC. However, the preliminary result of this study needs to be further confirmed with EPS produced by other *B. lactis* strains to determine whether this effect is specific to strains. We should concern that EPS produced by various genera, species, and even strains may play different roles due to their different chemical compositions and molecular structures. Undoubtedly, future in vivo studies and chemical structure determination will be critical to ultimately realize the potential of *B. lactis*-derived EPS in modulating gut autophagy, thereby preventing diarrhea induced by EPEC and maintaining intestinal mucosal physiology.

## 5. Conclusions

The results of the study proved the protective functions of EPS from *B. lactis* by its effects on facilitating autophagy flux, including enhancing autophagosome formation and degradation. EPS promoted autophagosome degradation by strengthening lysosomal function including reducing lysosomal pH and ascending lysosome-related protein expressions. The stimulation of autophagy flux mitigated excessive apoptosis, then ameliorated EPEC induced cell morphological changes and cell viability decrease in IPEC-J2 cells. Our findings suggest that the *B. lactis* EPS could be used to prevent EPEC-associated diarrhea, and also reveal the possible mechanism by which *B. lactis* plays a protective role through EPS.

## Data Availability

Data are available upon reasonable request from the corresponding author.
